# Cytochrome P4501A1 polymorphism as a susceptibility factor for breast cancer in postmenopausal Chinese women in Taiwan

**DOI:** 10.1038/sj.bjc.6690608

**Published:** 1999-08

**Authors:** C-S Huang, C-Y Shen, K-J Chang, S-M Hsu, H-D Chern

**Affiliations:** Departments of Surgery and Pathology and The Graduate Institute of Pharmaceutical Sciences, College of Medicine, National Taiwan University, Sec. 1, Taipei, Taiwan; Institute of Biomedical Sciences, Academia Sinica, Taipei, Taiwan

**Keywords:** CYP1A1, genetic polymorphism, susceptibility, breast cancer

## Abstract

The incidence of breast cancer has been greatly increasing in Taiwan over the past two decades. Since cytochrome P4501A1 (CYP1A1) is involved in the metabolism of environmental carcinogens or oestrogen, we hypothesized that CYP1A1 genetic polymorphism may be a susceptibility factor for breast cancer. This hypothesis was evaluated in this case control study of 150 breast cancer patients and 150 healthy controls among Chinese women. Two CYP1A1 polymorphisms were studied, one containing a new *Msp*1 site and the other located in axon 7 and resulting in the replacement of an isoleucine (Ile) residue by a valine (Val). After simultaneously considering the known or significant risk factors for breast cancer, including the age of study participants, positive family history of breast cancer, early menarche (≤ 13 years), nulliparity and late first full-term pregnancy (≥ 30 years), hormone replacement therapy and smoking, the CYP1A1 *Msp*1 polymorphism was found to be a significant factor in determining the risk of breast cancer. The homozygous variant was the most susceptible genotype with an adjusted odds ratio of 1.98 (95% confidence interval (CI) = 1.01–3.99) compared with the non-homozygous variants (the homozygous wild-type and the heterozygous variant). In contrast, the CYP1A1 Ile/Val polymorphism was not significantly associated with breast cancer development (adjusted OR = 1.07, 95% CI = 0.64–1.78). Interestingly, the *Msp*1 polymorphism was especially significant in postmenopausal women, but not in premenopausal women. Further stratification analysis in postmenopausal women who were non-smokers and with no history of hormone replacement therapy showed the cancer risk due to the *Msp*1 variant to be more significant in women with early menarche. We conclude that CYP1A1 polymorphism is a susceptibility factor for breast cancer in postmenopausal Chinese women in Taiwan. Further study with a large sample size should be considered to address issues of interactions between CYP1A1 and other risk factors. © 1999 Cancer Research Campaign


*British Journal of Cancer* (1999) **80**(11), 1838–1843
© 1999 Cancer Research Campaign
Article no. bjoc.1999.0608

### Cytochrome P4501A1 polymorphism as a susceptibility factor for breast cancer in postmenopausal Chinese women in Taiwan

**C-S Huang[1], C-Y Shen[4], K-J Chang[1], S-M Hsu[3] and H-D Chern[3]**

Departments of [1]Surgery and [2]Pathology and [3]the Graduate Institute of Pharmaceutical Sciences, College of Medicine, National Taiwan University, No. 1 Jen-Ai Road, Sec. 1, Taipei, Taiwan; [4]Institute of Biomedical Sciences, Academia Sinica, Taipei, Taiwan

**Summary** The incidence of breast cancer has been greatly increasing in Taiwan over the past two decades. Since cytochrome P4501A1 (CYP1A1) is involved in the metabolism of environmental carcinogens or oestrogen, we hypothesized that CYP1A1 genetic polymorphism may be a susceptibility factor for breast cancer. This hypothesis was evaluated in this case control study of 150 breast cancer patients and 150 healthy controls among Chinese women. Two CYP1A1 polymorphisms were studied, one containing a new *Msp*1 site and the other located in axon 7 and resulting in the replacement of an isoleucine (Ile) residue by a valine (Val). After simultaneously considering the known or significant risk factors for breast cancer, including the age of study participants, positive family history of breast cancer, early menarche (≤ 13 years), nulliparity and late first full-term pregnancy (≥ 30 years), hormone replacement therapy and smoking, the CYP1A1 *Msp*1 polymorphism was found to be a significant factor in determining the risk of breast cancer. The homozygous variant was the most susceptible genotype with an adjusted odds ratio of 1.98 (95% confidence interval (CI) = 1.01–3.99) compared with the non-homozygous variants (the homozygous wild-type and the heterozygous variant). In contrast, the CYP1A1 Ile/Val polymorphism was not significantly associated with breast cancer development (adjusted OR = 1.07, 95% CI = 0.64–1.78). Interestingly, the *Msp*1 polymorphism was especially significant in postmenopausal women, but not in premenopausal women. Further stratification analysis in postmenopausal women who were non-smokers and with no history of hormone replacement therapy showed the cancer risk due to the *Msp*1 variant to be more significant in women with early menarche. We conclude that CYP1A1 polymorphism is a susceptibility factor for breast cancer in postmenopausal Chinese women in Taiwan. Further study with a large sample size should be considered to address issues of interactions between CYP1A1 and other risk factors.

**Keywords:** CYP1A1; genetic polymorphism; susceptibility; breast cancer

Breast cancer formation is considered to be multifactorial, involving both genetic and environmental components. The effect of environmental factors, which has been shown in migrant studies (Ziegler et al, 1993), include diet (Hunter and Willett, 1993) or polyaromatic hydrocarbons (PAHs) (Li et al, 1996). PAHs from smoking or environmental sources can accumulate in breast and be metabolized to active carcinogens, which may contribute to breast carcinogenesis (Perera et al, 1995). Differences in bioactivation or detoxification of carcinogens may therefore have a role in determining individual susceptibility to breast cancer.

Cytochrome P4501A1 (CYP1A1) is involved in metabolizing PAHs. The metabolic intermediate is electrophilic, and can form carcinogenic DNA adduct. In addition, CYP1A1 may be involved in breast cancer via an oestrogen-related mechanism. Oestradiol is metabolized by two competing pathways to form either inactive 2-hydroxyestrone or active 16α-hydroxyestrone (Ball et al, 1990); level of the latter is often elevated in breast cancer, and may be related to tumorigenesis (Fishman et al, 1984; Osborne et al, 1993). CYP1A1 polymorphism may affect the distribution of these metabolites and then determine susceptibility to cancer (Yager, 1996; Zhu and Conney, 1998). Two major changes causing CYP1A1 polymorphism are reported: the T-A to C-G transition in the non-coding 3′-flanking region, which introduces a new *Msp*1 restriction site, and the A-T to G-C transition in exon 7, resulting in the replacement of an isoleucine (Ile) by valine (Val). For breast cancer susceptibility, a study of African-American patients has found a positive association with the *Msp*1, but not with the Ile/Val polymorphism (Taioli et al, 1995). One Caucasian study revealed a slightly elevated risk with Ile/Val polymorphism in postmenopausal women (Ambrosone et al, 1995). In contrast, these associations can not be confirmed by other studies (Bailey et al, 1998; Ishibe et al., 1998). The association of CYP1A1 with breast cancer risk in Asian women has never been studied. Since ethnic differences have frequently been seen in the relationship between CYP1A1 polymorphism and cancer risk, it was of interest to evaluate this relationship in Asian women, who show the lowest incidence of breast cancer in the world.

Breast cancer incidence has increased about threefold in the past two decades in Taiwan. Exposure to Western lifestyles is thought to have a substantial impact on the risk (Yang et al, 1997). Furthermore, increasing exposure to environmental pollutants, due to economic development, has been noted in Taiwanese. Since

*Received 8 September 1998*
*Revised 21 January 1999*
*Accepted 24 February 1999*

*Correspondence to:* H-D Chern

CYP1A1 is involved in the metabolism of both environmental carcinogens and oestrogen, its polymorphism may contribute to individual susceptibility to breast cancer. This study was conducted to investigate such a hypothesis.

### MATERIALS AND METHODS

### Subjects, materials and questionnaire

One hundred and fifty female breast cancer patients and 150 healthy female controls who had given their informed consent were enrolled. All breast cancer patients had pathologically confirmed primary breast carcinoma, and all were diagnosed and treated at the National Taiwan University Hospital between January 1995 and June 1996. This sample of female patients constituted about 50% of all the women with breast cancer attending our breast cancer clinic during the study period; the remaining patients were excluded due to the lack of sufficient blood specimens. No significant differences were found in breast cancer risk factor between the women selected and not selected. The healthy controls were randomly selected from the health examination clinic at the same hospital during the same study period, and constituted about 10% of all women attending the clinic. No significant differences were found in terms of socio-economic status between those included and excluded. The control subjects received a 1.5-day comprehensive health examination, not sponsored by the national health insurance programme, and showed no evidence of breast cancer, any suspicious precancerous lesions of the breast, or other cancers. A thorough, structured questionnaire was completed by both groups to provide relevant information regarding risk factors of breast cancer. An experienced research nurse was assigned to obtain this information. Information collected included age at diagnosis, family history of breast cancer (first-degree relatives), previous history of breast biopsy, age at menarche and/or menopause, parity, age at first full-term pregnancy (FFTP), number of pregnancies, history of breast-feeding, use of oral contraceptives, hormone replacement therapy (HRT), history of drinking alcohol and smoking cigarettes, ethnic background, residence area, family income and education level. The body mass index (BMI) and menopausal status were also recorded. Women younger than 55 years who had undergone hysterectomy, but not bilateral oophorectomy, were classified as unknown in terms of their menopausal status. On the basis of average family income and educational level, both case and control groups showed a high degree of homogeneity, and almost all (> 95% of both cases and controls) represented a population of middle-class women in Taiwan.

A 10-ml sample of peripheral blood, collected in acetate–citrate dextrose, was obtained from each breast cancer patient prior to treatment and from each control subject; the buffy coats of these specimens were prepared immediately and stored at –80°C until extraction of the genomic DNA. Genomic DNA was obtained by conventional phenol–chloroform extraction, followed by ethanol precipitation and was stored at –20°C for genotype analyses.

### Determination of CYP1A1 genetic polymorphism

Genotyping of the CYP1A1 Msp1 polymorphism was successfully performed for 141 patients and 145 controls using the polymerase chain reaction-restriction fragment length polymorphism (PCR-RFLP) method (Kawajiri et al, 1990; Hayashi et al, 1991). In brief, PCR amplification of a 340-base DNA fragment containing an *Msp*1 restriction site was performed, using the primers 5′-TAGGAGTCTTGTCTCATGCCT-3′(C44) and 5′-CAGTGAA-GAGGTGTAGCCGCT-3′(C47). The reaction mixture contained approximately 0.5 μg of genomic DNA, 0.5 units of *Taq* polymerase, 20 pmol of the pair of primers and 200 μM of dNTPs in a total volume of 50 μl. PCR was performed at 95°C for 10 min for the initial denaturation, following by 35 cycles of denaturation at 95°C for 30 s, annealing at 55°C for 30 s and extension at 72°C for 1 min. An aliquot of 12.5 μl of PCR product was digested with *Msp*1, then electrophoresed on 3% agarose gels for 1 h, and stained with ethidium bromide. Since the *Msp*1 variant allele creates a new *Msp*1 restriction site in the PCR product, it was identified by the presence of two bands on the gel. Three different genotypes were defined for the individual polymorphisms, these being the homozygous wild-type (no *Msp*1 site in either allele, wt/wt), the heterozygous variant (an *Msp*1 site in one allele, wt/vt), and the homozygous variant (an *Msp*1 site in both alleles, vt/vt).

The Ile/Val polymorphism was analysed in 143 cases and 145 controls using the PCR-RFLP method described by Oyama et al 1995). The primers used were: 1A1S (5′-GAACTGC-CACTTCAGCTGTCT-3′) and 1A1AS *Hinc*II (5′-GAAAGAC-CTCCCAGCGGTCA-3′). The 1A1AS *Hinc*II has a mismatch at the third residue (T) from the 3′ end of the primer that introduces an adenine residue at the second position of codon 463 and creates a *Hinc*II site next to the Val polymorphism at codon 462 by further PCR as above. The PCR products were digested with *Hinc*II and electrophoresed on 3% agarose gels and three different genotypes were defined, consisting of the homozygous wild-type (Ile/Ile), the heterozygous variant (Ile/Val) and the homozygous variant (Val/Val).

### Statistical analysis

Individual risk factors were first examined for their relationship to the risk of breast cancer using the $\chi^2$ test or *t*-test; if necessary, the Mantel $\chi^2$ test for trends was used to examine the dose–response relationship for the breast cancer risk estimates of various categories of genetic polymorphism; if a significant result was obtained, they were further examined using a multivariate logistic regression model. To obtain a model with biological plausibility, we included established risk factors in the logistic models regardless of statistical significance: the age of study participants, positive family history of breast cancer, the age at menarche and the age at FFTP (Kelsey, 1993). Next, the contribution of CYP1A1 polymorphism on breast cancer risk was evaluated under the condition that established risk factors or other significant risk factors were determined. A backward elimination procedure (Kleinbaum et al, 1982) was used to select the optimal model. Unconditional logistic regression analyses were performed to estimate multivariate-adjusted ORs (aORs) and their 95% CIs. All *P*-values were two-tailed.

### RESULTS

Table 1 shows all putative risk factors of breast cancer investigated in the present study. The mean age (± standard deviation (s.d.)) of the 150 breast cancer patients was 49.57 ± 11.34 years (range 26–82), which was slightly lower than that of the healthy controls (51.11 ± 11.82 years; range 25–84) ($P > 0.05$). The patients and

© 1999 Cancer Research Campaign

**Table 1** Univariate and multivariate analyses of risk factors for breast cancer in breast cancer patients ($n$ = 150) and control subjects ($n$ = 150), Taiwan

| Characteristics | Case | Control | OR | $P$ | aOR (95% CI)[a] |
|---|---|---|---|---|---|
| Age, year, mean ± s.d | | | | | |
| | 49.57 ± 11.34 | 51.11 ± 11.82 | – | 0.25 | 0.98 (0.96–1.00) |
| Age, year, at menarche, mean ± s.d. | | | | | |
| | 14.23 ± 1.78 | 14.73 ± 1.86 | – | 0.02 | – |
| Menopausal status | | | | | |
| Premenopausal | 57 (42.5) | 55 (41.7) | 1.00 | 0.89 | NS |
| Postmenopausal | 77 (57.5) | 77 (58.3) | 0.96 | | |
| Family history of breast cancer in mother or sister | | | | | |
| No | 134 (96.4) | 128 (96.2) | 1.00 | 0.94 | 1.04 (0.25–4.23) |
| Yes | 5 (3.6) | 5 (3.8) | 0.96 | | |
| Age at menarche, year | | | | | |
| > 13 | 82 (59.0) | 95 (71.4) | 1.00 | 0.03 | 1.00 |
| ≤ 13 | 57 (41.0) | 38 (28.6) | 1.74 | | 1.80 (1.1–3.1) |
| No. of full-term pregnancy | | | | | |
| > 3 | 25 (18.0) | 49 (37.7) | 1.00 | 0.001 | NS |
| 1–3 | 100 (71.9) | 73 (56.2) | 2.68 | (trend test) | |
| Nulliparity | 14 (10.1) | 8 (6.1) | 3.43 | | |
| Age at first full-term pregnancy, year, or nulliparity | | | | | |
| < 30 | 102 (73.4) | 118 (88.7) | 1.00 | 0.001 | 1.00 |
| ≥ 30 or nulliparity | 37 (26.6) | 11 (11.3) | 3.89 | | 2.68 (1.4–5.4) |
| Breast-feeding | | | | | |
| Yes | 64 (46.4) | 92 (70.2) | 1.00 | 0.001 | NS |
| No | 74 (53.6) | 39 (29.8) | 2.73 | | |
| History of cigarette smoking | | | | | |
| Never | 131 (94.2) | 131 (98.5) | 1.00 | 0.06 | 1.00 |
| Ever | 8 (5.8) | 2 (1.5) | 4.00 | | 5.50 (1.3–38.2) |
| Use of hormone replacement therapy | | | | | |
| Never | 118 (86.1) | 126 (94.7) | 1.00 | 0.02 | 1.00 |
| Ever | 19 (13.2) | 7 (5.3) | 2.90 | | 1.39 (1.4–9.1) |
| Body mass index, kg/m$^2$ | | | | | |
| <25 | 114 (82.0) | 90 (67.7) | 1.00 | 0.01 | NS |
| ≥ 25 | 25 (18.0) | 43 (32.3) | 0.46 | | |
| Use of oral contraceptives | | | | | |
| Never | 117 (85.4) | 108 (81.8) | 1.00 | 0.43 | NS |
| Ever | 20 (14.6) | 24 (18.2) | 0.77 | | |

* [a]OR, adjusted odds ratio from unconditional logistic regression which included the following risk factors: the age of study participants, positive family history of breast cancer, the age at menarche, the age at FFTP or nulliparity, breast feeding, history of HRT and smoking status. NS, not statistically significant at $P$ = 0.05 in logistic regression; these factors were removed from the final model.

controls were very similar in terms of ethnic background and residence area ($P$ > 0.05). The controls had a slightly higher family income and education level than the patients, but the difference was not significant ($P$ > 0.05). Of the established or likely risk factors for breast cancer examined in this study, the following were found to be significantly associated with cancer risk by univariate analysis: young age at menarche, nulliparity or an older age at FFTP (≥ 30 years), fewer number of full-term pregnancies, no history of breast-feeding, a history of HRT, current or previous history of cigarette smoking and a lower BMI. Further multivariate analysis simultaneously taking into account established risk factors or significant risk factors determined by univariate analysis, revealed that a younger age at menarche, nulliparity or an older age at FFTP, a history of HRT, and no history of breast-feeding were independent risk factors with a significant OR (Table 1). Family history (in first-degree relatives) was not found to be a significant risk factor by univariate and multivariate analysis. A similar proportion of patients and controls (3.6% and 3.8%) reported a positive family history of breast cancer. As our controls were from the group asking for health examination not sponsored by national health insurance, they might represent a group of women showing more concern about their health. In an earlier (1993–1994) case control study of breast cancer risk factors in the same hospital using breast cancer patients as the cases and cancer-free patients from other wards, such as ophthalmology ward, as the controls, 3.43% of the 175 cases and 0.44% of the 457 controls reported a positive family history of breast cancer in their first degree relatives (Chie et al, 1997, 1998). In the present study, the non-significant result for family history as a breast cancer risk factor therefore might result from the high proportion of controls with a family history.

The frequency distributions of the different genotypes for the CYP1A1 *Msp*1 and Ile/Val polymorphisms are shown in Table 2. A few specimens were not included in the analyses because of the poor quality of the DNA. The proportion (> 10% of variant CYP1A1 *Msp*1 genotype in controls) of women harbouring variant genotype was much higher than that (< 5%) observed in Western women, which results in a higher statistical power to evaluate such association in our population. A trend of increasing risk for Ile/Val heterozygotes and Val/Val homozygotes was suggested, particularly in postmenopausal women, but was not statistically significant ($P$ for trend = 0.07). In contrast, a significant increase in the risk of developing breast cancer was observed in the variant CYP1A1 *Msp*1 genotypes in postmenopausal women ($P$ for trend = 0.04). On the basis of the observation that a non-significant OR for the group of heterozygous variant was found as compared

 *© 1999 Cancer Research Campaign*

**Table 2** Distribution of CYPIA1 genetic polymorphism between breast cancer patients and controls in Taiwan

| Genotype | No. of cases (%) | No. of controls (%) | OR (95% CI) | *P* for trend |
|---|---|---|---|---|
| **Total women** | | | | |
| *Msp*1 polymorphism | | | | |
| wt/wt | 49 (34.7) | 48 (33.1) | 1.00 (referent) | 0.25 |
| wt/vt | 60 (42.6) | 80 (55.2) | 0.73 (0.42–1.28) | |
| vt/vt | 32 (22.7) | 17 (11.7) | 1.84 (0.86–4.00) | |
| Ile/Val polymorphism | | | | |
| Ile/Ile | 71 (49.7) | 80 (55.2) | 1.00 (referent) | 0.70 |
| Ile/Val | 64 (44.8) | 53 (36.5) | 1.36 (0.81–2.27) | |
| Val/Val | 8 (5.5) | 12 (8.3) | 1.75 (0.26–2.12) | |
| **Premenopausal women** | | | | |
| *Msp*1 polymorphism | | | | |
| wt/wt | 27 (42.9) | 20 (31.3) | 1.00 (referent) | 0.59 |
| wt/vt | 25 (39.7) | 36 (56.3) | 0.51 (0.22–1.19) | |
| vt/vt | 11 (17.4) | 8 (12.4) | 1.02 (0.30–3.43) | |
| Ile/Val polymorphism | | | | |
| Ile/Ile | 34 (54.0) | 31 (48.4) | 1.00 (referent) | 0.14 |
| Ile/Val | 28 (44.4) | 25 (39.1) | 1.02 (0.46–2.25) | |
| Val/Val | 1 (1.6) | 8 (12.5) | 0.11 (0.01–1.00) | |
| **Postmenopausal women** | | | | |
| *Msp*1 polymorphism | | | | |
| wt/wt | 22 (28.2) | 28 (34.6) | 1.00 (referent) | 0.04 |
| wt/vt | 35 (44.9) | 44 (54.3) | 1.01 (0.47–2.20) | |
| vt/vt | 21 (26.9) | 9 (11.1) | 2.97 (1.03–8.72) | |
| Ile/Val polymorphism | | | | |
| Ile/Ile | 37 (46.3) | 49 (60.5) | 1.00 (referent) | 0.07 |
| Ile/Val | 36 (45.0) | 28 (34.6) | 1.70 (0.84–3.45) | |
| Val/Val | 7 (8.7) | 4 (4.9) | 2.32 (0.55–10.3) | |

**Table 3** Unconditional logistic regression analysis of multiple risk factors of breast cancer in Taiwan

| Risk factor | Comparison | OR | 95% CI |
|---|---|---|---|
| CYP1A1 Msp1 | vt/vt vs wt/vt+wt/wt | 1.98[a] | 1.01–3.99[a] |
| Age of study participant | ± 1 year | 0.98 | 0.96–1.00 |
| Family history of breast cancer | | | |
| | Yes vs No | 1.12 | 0.27–4.55 |
| Age at menarche | ≤13 vs > 13 | 1.82 | 1.06–3.18 |
| Age at FFTP or nulliparity | ≥30 or nulliparity vs < 30 | | |
| | | 2.59 | 1.32–5.27 |
| HRT | Yes vs No | 3.60 | 1.47–9.78 |
| Smoking | Yes vs No | 5.10 | 1.14–35.9 |

[a]The contribution of CYP1A1 Msp1 polymorphism to breast cancer development was adjusted by all known or significant risk factors included in the model (age, postive family history of breast cancer, age at menarche, age at FFTP or nulliparity, history of HRT and smoking status).

**Table 4** Unconditional logistic regression analysis of CYP1A1 Msp1 polymorphism in relation to breast cancer risk in postmenopausal women without a history of smoking or HRT

| Risk factor | Comparison | OR | 95% CI |
|---|---|---|---|
| CYP1A1 Msp1 | vt/vt vs wt/vt+wt/wt | 2.82* | 1.02–7.69[a] |
| Age at menarche | ≤ 13 vs > 13 | 2.53 | 1.14–5.64 |
| Age at FFTP or nulliparity | | | |
| | ≥ 30 or nulliparity vs <30 | 2.86 | 0.96–8.49 |

[a]The contribution of CYP1A1 Msp1 polymorphism to breast cancer development in postmenopausal women without a history of smoking or HRT was adjusted by the age at menarche and the age at FFTP or nulliparity.

to the group of homozygous wild-type, we classified the genotypes of the study subjects into two groups, homozygous variant and non-homozygous variant (consisting of the homozygous wild-type and the heterozygous variant) in the following analysis. The proportion of homozygous variants versus non-homozygous variants for the *Msp*1 polymorphism in patients with breast cancer was significantly higher than that in the control group (OR, 2.21; 95% CI 1.11–1.41) by univariate analysis. Interestingly, this association was more obvious in postmenopausal women (OR, 2.95; 95% CI 1.17–7.58), but not significant in premenopausal women. In contrast, the proportion of the homozygous variant of the Ile/Val polymorphism was not significantly different between breast cancer patients and healthy controls, though a slightly significant association was seen in postmenopausal women.

Multivariate logistic regression analysis was performed to assess the effect of CYP1A1 genetic polymorphism on breast cancer risk after adjusting for other established or significant risk factors, i.e. the age of study participants, positive family history of breast cancer, early menarche, nulliparity or an older age at FFTP, a history of HRT and smoking (Table 3). The results showed that the homozygous variant of the CYP1A1 *Msp*1 polymorphism remained a significant risk factor for breast cancer (aOR, 1.98; 95% CI 1.01–4.00). In contrast, the CYP1A1 Ile/Val polymorphism gave no correlation with breast cancer risk in the multivariate model (aOR, 1.07; 95% CI 0.64–1.78).

Further analysis, stratified by different risk factors, was performed to explore possible mechanisms of breast tumorigenesis related to CYP1A1. Since the number of women with a history of

© 1999 Cancer Research Campaign smoking or HRT was very small in this study (as it is in Taiwanese women in general), a multivariate logistic regression analysis of individual subgroups based on smoking or HRT status was not feasible, and we therefore restricted our analysis to post-menopausal women without any history of smoking or HRT (Table 4). We did not include the age of study participants and positive family history of breast cancer in the model, because that age distribution in these women was relatively homogenous and family history does not seem to be related to late-onset (post-menopausal) breast cancer. The results showed that CYP1A1 *Msp*1 polymorphism remained a significant risk factor (aOR, 2.82; 95% CI 1.02–7.69). Further stratification of this specific subgroup of women by age at menarche revealed that CYP1A1 polymorphism was particularly significant in women with early menarche, the risk of breast cancer being eight times as great in women with the homozygous variant as in those with the non-homozygous variants (95% CI 1.23–158). In contrast, this association was not obvious in women with late menarche ($P = 0.06$).

### DISCUSSION

Our study shows that a homozygous variant of the CYP1A1 *Msp*1 polymorphism was a significant risk factor associated with breast cancer, independent of other established risk factors. This study is the first to evaluate such an association in a Chinese population, having the lowest incidence (20 cases per 100 000 women per year) in the world (Lo et al, 1998). No association of the homozygous CYP1A1 Val/Val genotype and breast cancer risk was seen; this is consistent with the results of an in vitro study showing that the Ile and Val forms of CYP1A1 have a similar metabolic capacity to bioactivate carcinogens (Zhang et al, 1996).

Since CYP1A1 is involved in the activation of procarcinogens, it is reasonable to observe an association between CYP1A1 polymorphism and cancer risk, especially in cancers caused by chemical carcinogens. For example, it has frequently been reported that an increased risk of lung cancer is related to variant CYP1A1 *Msp*1 genotypes (Kawajiri et al, 1993; Kihara and Noda, 1995; Xu et al, 1996); however, the results vary among ethnic groups (Drakoulis et al, 1994; Hirvonen et al, 1992; Alexandrie et al, 1994). In breast cancer, a significant association related to *Msp*1 polymorphism was found in African-American patients (Taioli et al, 1995); however, this was not apparent in Caucasians (Bailey et al, 1998; Ishibe et al, 1998). These discrepancies in cancer risk associated with CYP1A1 variant genotypes may be explained, in part, by ethnic differences in the frequency of the genotypic polymorphism. Almost all studies assessing the relation between CYP1A1 and cancer are based on a case control design. Whether CYP1A1 polymorphism is significant in such a design depends on the frequency of variant genotypes in the controls as well as in the cases. The frequencies of a variant allele or genotype vary markedly between ethnic groups, being much higher in the Asian than in the Caucasian, with an intermediate value in the African-American. The significantly low prevalence of a variant allele and especially the homozygous variant genotype in Caucasians results in a lower statistical power for detecting an effect of CYP1A1 polymorphism on cancer risk. In contrast, in Asian studies, including our own, more than 10% of the study populations possess variant genotypes, and, therefore, adequate statistical power facilitates the evaluation of such an association.

Ethnic differences in CYP1A1 polymorphism related to cancer risk may also be attributable to difference in exposure ‘dosage’ to procarcinogens in the different populations. Specific genotypes conferring protection/susceptibility to cancer risk commonly reveal their protective/promoting effect in subjects exposed to a ‘normal dose’ of procarcinogens. For example, the greatest incremental lung cancer risk (sevenfold) for the ‘susceptible’ CYP1A1 genotype was seen in light smokers, whereas heavy smokers with this genotype had less than twice the risk of heavy smokers without the genotype (Nakachi et al, 1991). A study also showed that the increased level of carcinogen-DNA adduct related to genetic polymorphism was seen only in individuals exposed to levels of procarcinogens below the average level, while adduct levels in individuals exposed to higher levels were similar, regardless of the genetic status of their susceptibility genes (Kato et al, 1995). Thus, the significant effect seen with CYP1A1 in relation to breast cancer risk in our population, but not in Caucasian populations, might be due to the relatively lower levels of procarcinogens among Taiwanese women. Supporting this hypothesis, it is notable that Asian women have, on average, 20% lower serum oestradiol levels than Western women (Bernstein et al, 1990).

In this study, the association between CYP1A1 polymorphism and breast cancer was more striking in postmenopausal women. Since carcinogenesis requires an extended period to accumulate genetic mutations, the promoting effect of carcinogens, including oestrogen, may affect the breast tissue in the premenopausal period and contribute to the development of postmenopausal breast cancer. Thus, an increased cancer risk related to CYP1A1 genotypes in postmenopausal women may be due to the long period of time required for CYP1A1-related mechanisms to accumulate their carcinogenic effect. Our observation that the CYP1A1-*Msp*1 genotype-related susceptibility to breast cancer was also more evident in women over 50 years of age (OR in women > 50 years, 2.67, $P = 0.03$ vs OR in women ≤ 50 years, 1.73, $P = 0.24$), regardless of their menopausal status, supports this idea.

PAHs and oestrogen are two CYP1A1-related procarcinogens. However, our analysis focusing on postmenopausal women without any history of smoking or HRT still showed a significantly increased risk posed by the CYP1A1 variant. This finding suggests that, for these women, CYP1A1-related carcinogen might be from source other than smoking and HRT. Whether these undefined carcinogens are related to increasing amount of pollutants, including environmental oestrogen (such as pesticides) or PAHs (from many air pollution sources) due to economic boom in Taiwan, is an interesting hypothesis remaining to be explored. Furthermore, in postmenopausal women without smoking and HRT, the breast cancer risk related to CYP1A1 was more obvious in women with early menarche. Women with early menarche had been exposed to estrogen for a longer period of time, which might amplify the effect of the CYP1A1 variants. However, due to limited sample size of these subgroups in this study, these interpretations should be confirmed by further research.

Our study shows that the CYP1A1 *Msp*1 polymorphism is a significant susceptibility factor for breast cancer among post-menopausal Chinese women in Taiwan. For a population with a relatively higher frequency of the homozygous variants of CYP1A1 *Msp*1, such findings not only provide clues toward understanding the role of CYP1A1-related carcinogens during breast tumorigenesis, but are also of special public health importance. Although CYP1A1 poses a low individual risk, it is not an uncommon genetic trait and could be an important determinant of population risk.

© 1999 Cancer Research Campaign

### ACKNOWLEDGEMENTS

We are grateful to Drs Ming-Whei Yu (National Taiwan University), David B Thomas (Fred Hutchinson Cancer Research Center) and Timothy R Rebbeck (University of Pennsylvania) for valuable suggestions and criticisms. This study was supported by the National Science Council of Taiwan, Grant NSC 8602314–B002–176.

### REFERENCES

Alexandrie AK, Sundberg MI, Seidegard J, Torniling G and Rannug A (1994) Genetic susceptibility to lung cancer with special emphasis on CYP1A1 and GSTM1: a study on host factors in relation to age at onset, gender and histological cancer types. *Carcinogenesis* **15**: 1785–1790

Ambrosone CB, Freudenheim JL, Graham S, Marshall JR, Vena JE, Brasure JR, Laughlin R, Nemoto T, Michalek AM, Harrington A, Ford TD and Shield PG (1995) Cytochrome P4501A1 and glutathione S-transferase (M1) genetic polymorphism and postmenopausal breast cancer risk. *Cancer Res* **55**: 3483–3485

Bailey LR, Roodi N, Verrier CS, Yee CJ, Dupont WD and Parl FF (1998) Breast cancer and CYP1A1, GSTM1 and GSTT1 polymorphism: evidence of a lack of association in Caucasians and African Americans. *Cancer Res* **58**: 65–70

Ball SE, Forrester LM, Wolf CR and Back DJ (1990) Differences in the cytochrome P450 isoenzymes involved in the 2-hydroxylation of estradiol and 17α-ethinylestradiol. *Biochem J* **267**: 221–226

Bernstein L, Yuan JM, Ross RK, Pike MC, Hanisch R, Lobo R, Stanczyk F, Gao YT and Henderson BE (1990) Serum hormone levels in pre-menopausal Chinese women in Shanghai and white women in Los Angeles: results from two breast cancer case-control studies. *Cancer Causes Control* **1**: 51–58

Chie WC, Lee WC, Li CY, Huang CS, Chang KJ, Yen ML and Lin RS (1997) Lactation, lactation suppression hormones and breast cancer: a hospital-based case-control study for parous women in Taiwan. *Oncol Rep* **4**: 319–326

Chie WC, Li CY, Huang CS, Chang KJ and Lin RS (1998) Body size factors of different ages and breast cancer risk in Taiwan. *Anticancer Res* **18**: 565–570

Drakoulis N, Cascorbi I, Brockmoller J, Gross CR and Roots I (1994) Polymorphism in the human CYP1A1 gene as susceptibility factors for lung cancer: exon-7 mutation (4889 A to G), and a T to C mutation in the 3′-flanking region. *Clin Invest* **72**: 240–248.

Fishman J, Schneider J, Hershcope RJ and Bradlow HL (1984) Increased estrogen-16α-hydroxylase activity in women with breast and endometrial cancer. *J Steroid Biochem* **20**: 1077–1081

Hayashi SI, Watanabe J, Nakachi K and Kawajiri K (1991) Genetic linkage of lung cancer-associated Msp1 polymorphism with amino acid replacement in the heme binding region of the human cytochrome P4501A1 gene. *J Biochem* **110**: 407–411

Hirvonen A, Husgafvel-Pursiainen K, Karjalainen A, Anttila S and Vainio H (1992) Point-mutational Msp1 and Ile-Val polymorphism closely linked in the CYP1A1 gene: lack of association with susceptibility to lung cancer in a Finnish study population. *Cancer Epidemiol Biomarkers Prev* **1**: 485–489

Hunter DJ and Willett WC (1993) Diet, body size, and breast cancer. *Epidemiol Rev* **15**: 110–132

Ishibe N, Hankinson Se, Colditz GA, Spiegelman D, Willett WC, Speizer FE, Kelsey KT and Hunter DJ (1998) Cigarette smoking, cytochrome P4501A1 polymorphism, and breast cancer risk in the nurses health study. *Cancer Res* **58**: 667–671

Kato S, Bowman ED, Harrington AM, Blomeke B and Shields PG (1995) Human lung carcinogen-DNA adduct levels mediated by genetic polymorphism in vivo. *J Natl Cancer Inst* **87**: 902–907

Kawajiri K, Nakachi K, Imai K, Yoshii A, Shinoda N and Watanabe J (1990) Identification of genetically high risk individuals to lung cancer by DNA polymorphism of the cytochrome P4501A1 gene. *FEBS Lett* **263**: 131–133

Kawajiri K, Nakachi K, Imai K, Watanabe J and Hayashi S (1993) The CYP1A1 gene and cancer susceptibility. *Crit Rev Oncol Hematol* **14**: 77–87

Kelsey JL (1993) Breast cancer epidemiology: summary and future directions. *Epidemiol Rev* **15**: 36–47

Kihara M and Noda K (1995) Risk of smoking for squamous and small cell carcinomas of the lung modulated by combinations of CYP1A1 and GSTM1 gene polymorphism in a Japanese population. *Carcinogenesis* **16**: 2331–2336

Kleinbaum DG, Kupper LL and Morgenstern H (1982) *Epidemiologic Research*. Van Nostrand Reinhold: New York

Li D, Wang M, Dhingra K and Hittelman WN (1996) Aromatic DNA adducts in adjacent tissues of breast cancer patients: clues to breast cancer etiology. *Cancer Res* **56**: 287–293

Lo YL, Yu JC, Huang CS, Tseng SL, Chang TM, Chang KJ, Wu CW and Shen CY (1998) Allelic loss of the *BRCA1* and *BRCA2* genes and other regions on 17q and 13q in breast cancer among women from Taiwan (area of low incidence but early onset). *In J Cancer* **79**: 580–587

Nakachi K, Imai K, Hayashi S, Watanabe J and Kawajiri K (1991) Genetic susceptibility to squamous cell carcinoma of the lung in relation to cigarette smoking dose. *Cancer Res* **51**: 5177–5180

Osborne MP, Bradlow HL, Wong GY and Telang NT (1993) Upregulation of estradiol C16α-hydroxylation in human breast tissue: a potential biomarker of breast cancer risk. *J Natl Cancer Inst* **85**: 1917–1920

Oyama T, Mitsudomi T, Kawamoto T, Ogami A, Osaki T, Kodama Y and Yasumoto K (1995) Detection of CYP1A1 gene polymorphism using designed RFLP and distributions of CYP1A1 genotypes in Japanese. *Int Arch Occup Environ Health* **67**: 253–256

Perera FP, Estabrook A, Hewer A, Channing K, Rundle A, Mooney LA, Whyatt R and Phillips DH (1995) Carcinogen-DNA adducts in human breast tissue. *Cancer Epidemiol Biomarkers Prev* **4**: 233–238

Taioli E, Trachman J, Chen X, Toniolo P and Garte SJ (1995) A CYP1A1 restriction fragment length polymorphism is associated with breast cancer in African-American women. *Cancer Res* **55**: 3757–3758

Yager JD (1996) Molecular mechanisms of estrogen carcinogenesis. *Annu Rev Pharmacol Toxicol* **36**: 203–232

Yang PS, Yang TL, Liu CL, Wu CW and Shen CY (1997) A case-control study of breast cancer in Taiwan: a low incidence area. *Br J Cancer* **75**: 752–756

Xu X, Kelsey KT, Wiencke JK, Wain JC and Christiani DC (1996) Cytochrome P450 CYP1A1 *Msp*1 polymorphism and lung cancer susceptibility. *Cancer Epidemiol Biomarkers Prev* **5**: 687–692

Zhang ZY, Fasco MJ, Huang L, Guengerich FP and Kaminsky LS (1996) Characterization of purified human recombinant cytochrome P4501A1-Ile and -Val462: Assessment of a role for the rare allele in carcinogenesis. *Cancer Res* **56**: 3926–3933

Zhu BT and Conney AH (1998) Functional role of estrogen metabolism in target cells: review and perspectives. *Carcinogenesis* **19**: 1–27

Ziegler RG, Hoover RN, Pike MC, Hildesheim A, Nomura AM, West DW, Wu-Williams AH, Kolonel LN, Horn-Ross PL, Rosenthal JF and Hyer MB (1993) Migration patterns and breast cancer risk in Asian-American women. *J Natl Cancer Inst* **85**: 1819–1827

© 1999 Cancer Research Campaign
